# Benefits of Integrating Standardized Patient Encounters into Pre-clerkship Neurology Curriculum

**DOI:** 10.1007/s40670-026-02801-9

**Published:** 2026-06-29

**Authors:** Hope Cherian, Rachel Dockter, Emiri Uchiyama, Jenny Fortun

**Affiliations:** Wertheim College of Medicine, Florida International University Herbert, 11200 SW 8th St AHC2, Miami, FL 33199 USA

**Keywords:** Standardized patient encounters, Pre-clerkship medical education, Neuroscience education, Knowledge acquisition

## Abstract

**Introduction:**

Standardized patient encounters (SPEs) are a foundational tool in medical education, yet their pre-clerkship role in undergraduate medical education, particularly in neuroscience, remains less clearly defined. This study evaluates the impact of integrating SPEs into a pre-clerkship Nervous System course on student knowledge, diagnostic confidence, and performance. Our objective was to design reproducible neurology-focused SPEs for pre-clerkship learners and assess its effectiveness in improving knowledge acquisition and diagnostic confidence with common neurological conditions.

**Methods:**

Second-year medical students at our institution who were enrolled in a 6-week Nervous System course participated in four structured SPEs addressing common neurologic presentations. Students conducted focused neurologic histories, examinations, and diagnostic reasoning. Knowledge was assessed using a 10-item quiz administered before and after the SPEs. Performance on 12 SPE-related NBME final exam questions was compared with national averages. Diagnostic confidence was evaluated using pre- and post-SPE self-assessment surveys.

**Results:**

Students demonstrated significant improvement in knowledge, with a median quiz score increase of 1.0 point (Hodges-Lehmann estimate, 95% CI: 0.0-3.5; *p* < 0.0001). On the NBME exam, our students outperformed national averages across nearly all SPE-related items (FIU mean = 0.87 vs. 0.71; *p* < 0.05). Students also reported significant increases in diagnostic confidence across all neurologic conditions assessed (all *p* < 0.001).

**Discussion:**

Integration of SPEs into a pre-clerkship neurology course resulted in immediate gains in knowledge and confidence, and higher standardized exam performance, underscoring SPEs as a valuable early experiential learning modality in clinical neuroscience education.

## Introduction

Neuroscience and neurology have long been challenging areas in medical education, partially due to the topics’ complexity and limited early clinical exposure to anchor foundational knowledge [1]. The resulting students’ apprehension towards neurology may hinder learning, underscoring the need for effective transformation of pre-clerkship medical education. We have previously reported that incorporating active learning sessions and patient encounters in the pre-clerkship neurology curriculum supports learning and contributes to a richer learning experience [2, 3].

Standardized patient encounters (SPEs) may be an additional yet unexplored tool to bridge classroom learning with clinical application. SPEs offer learners structured opportunities to apply knowledge, practice clinical skills and real-time critical thinking in controlled settings, while receiving immediate feedback and practicing professionalism, communication, and collaboration, all which are essential for future practice [4]. Most SPE literature demonstrating enhanced knowledge and communication skills focuses on students in the clerkship phase or at the graduate level as residents and fellows [5–9]. Participating in SPEs has shown clear benefits during the neurology clerkship, including improved National Board of Medical Examiner (NBME) exam scores [10], self-confidence in their performance of managing acute emergencies [11], and more favorable learner perceptions [12]. Similarly, simulation training was shown to improve residents’ and fellows’ performance in multiple neurologic cases, including acute stroke management [13, 14] and guideline adherence for status epilepticus [15]. Importantly, SPEs enhance confidence, which is associated with improved performance in clinical settings [16].

Despite the widespread use of SPEs in the clinical years, their integration into pre-clerkship education is rather limited and focused on student perceptions (Kirkpatrick 1) rather than objective score improvement (Kirkpatrick 2) [17, 18]. In one study, implementation of SPE during the pre-clerkship phase was associated with improved neurological examination performance in the fourth year of medical school [19]. Grounded in experiential learning theory, which emphasizes the role of concrete experience, reflective observation, and active experimentation in knowledge acquisition [20], we introduced SPE in the pre-clerkship curriculum. By engaging learners in structured feedback-rich environment earlier in their training, SPEs may facilitate the integration of foundational concepts with clinical reasoning, promoting deeper learning. Thus, we hypothesized that structured SPE introduced early in the pre-clerkship curriculum would enhance learning and confidence. This study describes the design and implementation of SPE sessions in the pre-clerkship neuroscience curriculum, highlighting best practices in design and implementation, and evaluates their effectiveness with respect to knowledge gains and confidence diagnosing common neurologic conditions.

## Methods

### Participants and Setting

This study was conducted during the second-year Nervous System course at Florida International University, Herbert Wertheim College of Medicine. All second-year medical students enrolled in the course (*N* = 121, academic year 2024–2025) participated in this study.

### Curriculum Description & Design

The Nervous System is a second-year 8-week course that consists of lectures, case discussions, and laboratory sessions. The SPE sessions were implemented for the first time in 2024 as part of the standard curriculum during the last week of the course.

Students completed the SPE sessions in small groups of 7–8, rotating through four 25-minute encounters (each student participated for 2 h). To maintain small group size, the class was split in half and the SPE was duplicated, resulting in a total of 4 instructional hours out of 116 curricular hours for the course. During each SPE, groups were expected to obtain a focused history, perform a targeted neurological examination, develop a differential diagnosis, propose and interpret diagnostic tests, and integrate clinical findings with the relevant neuroanatomy and pathophysiology. To ensure high fidelity and consistency across groups, we used a standardized case script and facilitator guide. Several cycles of structured feedback from faculty and standardized patients (SPs) informed iterative refinements to the script and facilitator guide. The SPE design was integrative, incorporating elements of group discussion, communication and skills practice. All SPE were facilitated by a faculty, who used a standard guide to facilitate the discussion and a grading rubric to provide formative feedback to students and standardize expectations. This rubric was not used for summative assessment and did not include an in-depth evaluation of the different elements of SPE on learning. Other than real-time individualized formative feedback, communication and other non-technical skills were not formally assessed as study outcomes because not all students performed all tasks. Standardized patients also provided formative feedback but were not involved in summative assessment of student performance.

**Fig. 1:**
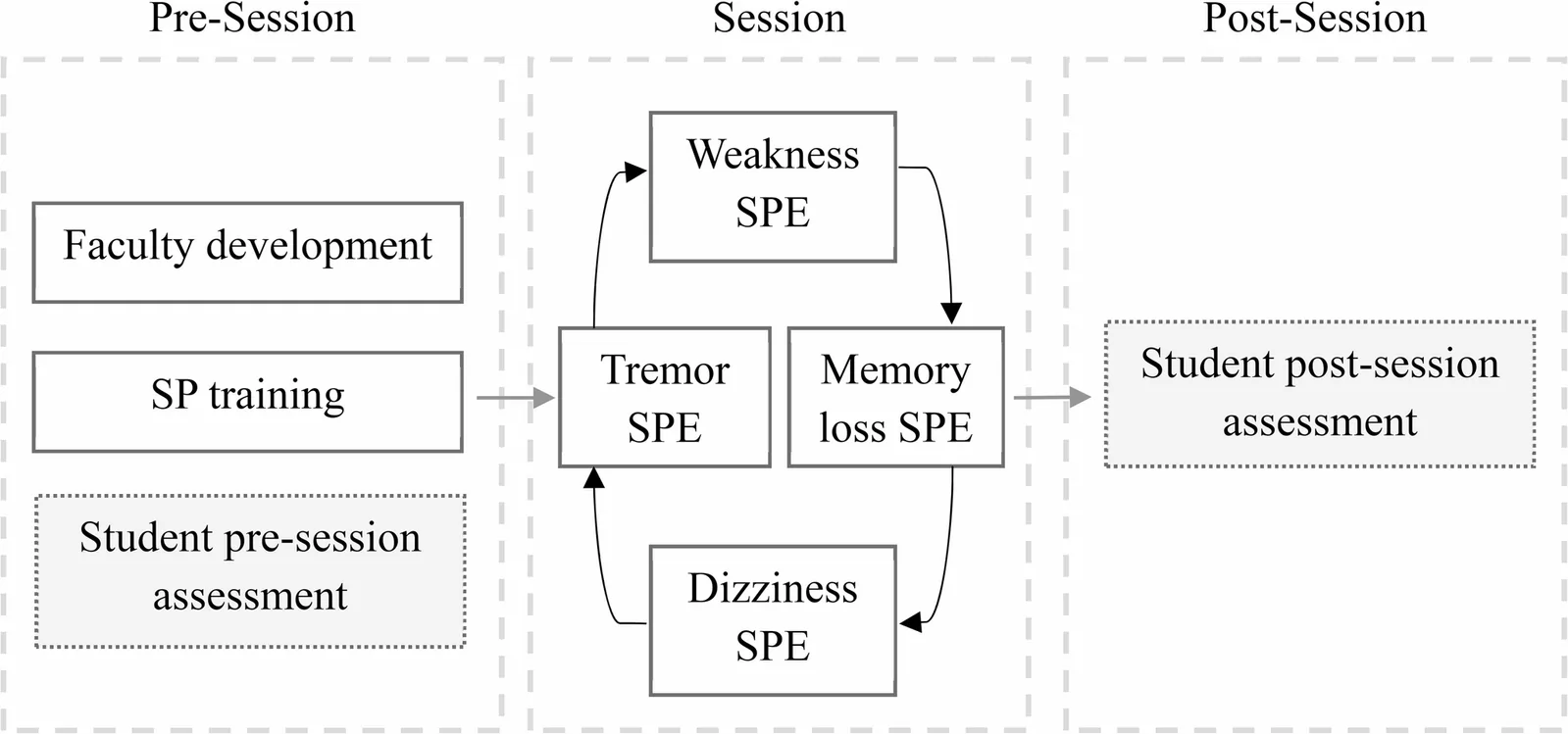
Overview of the standardized patient encounter (SPE) instructional sequence. Faculty development sessions and standardized patient (SP) training occurred prior to the educational activity to ensure consistent facilitation and accurate portrayal of neurologic cases. Before the session, students completed the student pre-session assessment (highlighted in the figure), which included a voluntary, ungraded knowledge quiz and baseline self-reported confidence questions. During the session, small groups of 7–8 students rotated through four 25-minute SPE stations (tremor, weakness, memory loss, and dizziness), allowing every student to experience all cases. At the conclusion of the session, students completed the student post-session assessment (also highlighted), which included the same knowledge-based and confidence items. Abbreviations. SP: Standardized Patient; SPE: Standardized Patient Encounter

A summary of the instructional sequence can be found in Fig. 1.

Each case was developed collaboratively by the course director, neurology faculty, and the SP Program with scripts including medical history and neurological examination expectations. We used a targeted approach to select cases and topics for discussion, focusing on learning gaps and common neurological presentations that were previously identified by students and teaching faculty as challenging during the end-of-course evaluations. The four presentations selected were tremors, weakness, memory loss, and vertigo. The final diagnoses were essential tremor, Guillain-Barré Syndrome, vascular dementia, or benign paroxysmal positional vertigo. Although there were only four diagnoses portrayed by the SPs, a group of differential diagnoses were also explicitly discussed as part of the relevant presentations – Alzheimer’s disease, Parkinson’s disease, Lewy body dementia, cerebellar tremor, vestibular neuritis, and Ménière’s disease. The SPE content represented 4 of 12 symptom categories and 11 of 72 diagnoses covered in the course.

The diagnoses discussed in the SPE session were previously covered in the course, but not in prior courses. Students were not expected to prepare for the session and were not aware of the cases or diagnoses that the session would cover. They were informed that an ungraded voluntary quiz would be administered and were encouraged to take it by offering a bonus point for completion.

### Standardized Patient Recruitment and Training

Paid SPs were recruited from the institution’s established SP program. Training for the SPs consisted of orientation, case preparation, and examination portrayal training. The orientation gave an overview of course objectives, professional expectations, and encounter logistics. Case preparation reviewed case scripts including patient history, affect, and anticipated student questions. Examination portrayal training covered standardized responses to neurological examination maneuvers and coaching in simulating examination findings. Calibration was achieved through faculty-led pilot sessions to ensure consistency and fidelity.

### Faculty Preparation

Faculty members serving as facilitators during the SPEs participated in a structured 1-hour development workshop designed to align teaching with course goals. The workshop addressed session logistics, standardization of case facilitation across groups, and strategies for reinforcing basic science principles during clinical encounters. Faculty facilitators were instructed to observe group performance and provide feedback, focusing on guiding the group discussion rather than providing answers or explanations. All SPs participated in this additional workshop.

### Outcomes and Analysis

The primary outcome of this study was knowledge gain, with a secondary outcome of student confidence.

Knowledge gain was evaluated by comparing the scores in an identical 10-item multiple choice quiz administered prior to (pre) and following (post) the session. The quiz was developed by the course director and reviewed through a structured, multi-step process to ensure that each item was accurate, clear, aligned with activity goals, and measured intended outcomes. Two content experts and a copy editor participated in this review. Students did not receive grades or feedback after the pre-session quiz and were not aware that the post-session quiz would be identical. Additionally, the score on 12 SPE-related questions included in the end-of-course exam was compared against the historical performance data as the national benchmark. The end-of-course exam consisted of questions from the Customized Assessment Service (CAS) program through the National Board of Medical Examiners (NBME) and was administered on the final day of the course. For this exam, the course director selected questions that reflected SPEs’ learning outcomes and mapped each question to the diagnosis explicitly covered in each station. NBME CAS questions were tagged with topic (diagnosis), type of question and lead-in. Types of questions included higher-order skills (application, analysis); lead-in were subdivided into diagnosis (final SPE and SPE differential), management, and underlying mechanisms. The aggregated p-value of each question was provided by NBME and included the school’s p-value (our students’ performance in each question) and historical performance data, which was used as an external benchmark for comparison. Item-level statistical comparisons with national NBME data were not feasible, as only aggregate performance metrics were available.

Students’ confidence in diagnosing the targeted neurological conditions was evaluated using a three-point Likert scale (1 = not confident, 2 = neutral, 3 = confident). Questions were included at the end of the pre- and post-quiz.

Statistical analysis: Data was anonymized for analysis. Data on quizzes and the final exam were not normally distributed, as confirmed by the Shapiro-Wilk normality test. Individual differences between pre- and post-session quiz (knowledge and confidence) were evaluated using a Wilcoxon signed-rank test, and differences between our students’ NBME performance and national grouped performance were evaluated using a Mann-Whitney U test. We excluded 38 confidence ratings out of 2,530 total, due to missing or multiple answer responses. Statistical significance was defined as a p-value of less than 0.05. Statistical analysis and graphing were performed in Python 3.13.

### Standard Protocol Approvals, Registrations, and Patient Consents

The format of SPE sessions, examinations, and surveys were all part of the standard curriculum work. This project was determined to be exempt by the Institutional Review Borad at Florida International University, which granted approval (IRB-22-0472-AM01 #112236).

### Data Availability

Data will be made available through request directed to the corresponding author.

## Results

To evaluate the effectiveness of SPEs in the pre-clerkship Nervous System course, we examined knowledge and confidence.

**Fig. 2:**
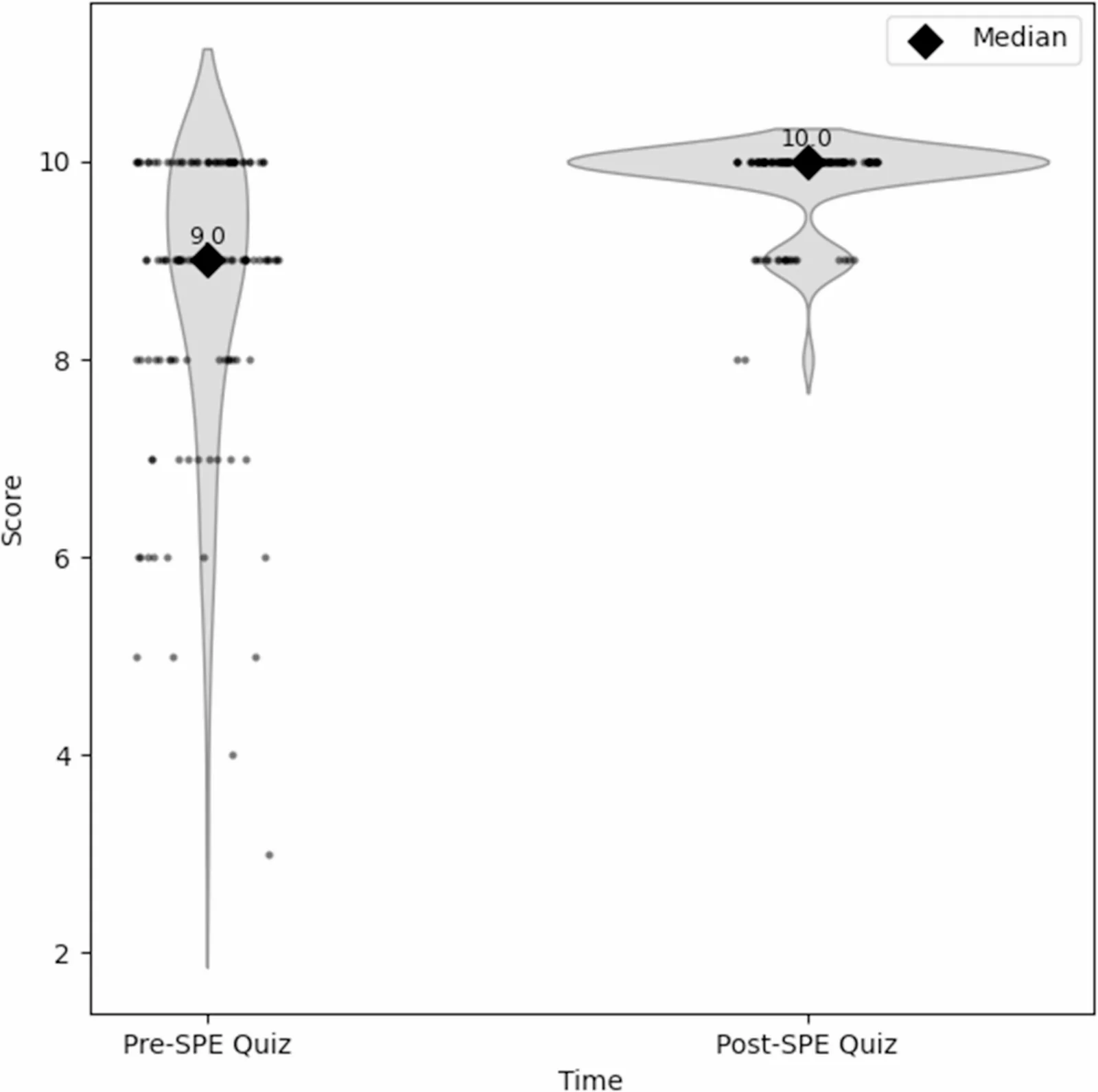
Distribution of pre- and post-standardized patient encounter (SPE) knowledge-based quiz scores. Each point represents an individual student’s score. Violin plots display the kernel-density distribution for pre-SPE and post-SPE quiz performance, with overlaid beeswarm points to illustrate individual paired observations. The plot demonstrates the overall shift in score distributions before and after the standardized patient encounter (SPE). The median increase in quiz score from before and after the session was 1.0 point (Hodges-Lehmann estimate, 95% CI: 0.0-3.5)

Figure 2 illustrates the distribution of pre- and post-quiz scores. Compared to the pre-quiz, the post-quiz scores centered at higher values with a narrowed spread, suggesting overall improvement and reduced variability. The median increase in quiz scores pre- to post-session was 1.0 out of 10 points (Hodges-Lehmann estimate, 95% CI: 0.0-3.5; V = 63.0, *p* < 0.0001).

Table 1 compares average scores on SPE-related questions in the final exam between students at our college and national NBME averages. Our students significantly outperformed the national average across nearly all topics (College mean = 0.87 vs. NBME mean = 0.71; *p* < 0.05). The largest differences between the 2 cohorts were observed in questions related to Ménière’s Disease (+ 41% points), Benign Paroxysmal Positional Vertigo (+ 22), and Essential Tremor (+ 36). The effect size, expressed as the rank-biserial correlation, was 0.57, indicating that our scores tended to be higher than National scores. Of note, there were no differences between scores in SPE-final diagnoses and differential diagnoses discussed in the session for our students (0.91 vs. 0.84; *p* > 0.05). Additionally, the reported NBME median of these students (SPE cohort, 0.83 current cohort, nationally reported item difficulty of 0.75) was not different than a prior cohort in a similar NBME exam (p-value, 0.84, nationally reported item difficulty of 0.76).

**Table 1:** Average score on NBME final examination questions, by group

Question Topic			Type	Lead- in	College	National^
Essential tremor*			Apply	Diagnosis	0.77	0.57
Essential tremor*			Apply	Management	0.96	0.45
Guillain-Barré Syndrome*			Apply	Diagnosis	0.98	0.89
Vascular dementia*			Apply	Diagnosis	0.97	0.82
Benign Paroxysmal Positional Vertigo*			Apply	Diagnosis	0.89	0.67
Alzheimer’s disease			Analyze	Pathophysiology	0.87	0.88
Alzheimer’s disease			Analyze	Pathophysiology	0.93	0.76
Ménière’s disease			Apply	Diagnosis	0.96	0.55
Parkinson’s disease			Analyze	Pathophysiology	0.62	0.54
Parkinson’s disease			Apply	Management	0.62	0.56
Frontotemporal dementia			Apply	Diagnosis	0.89	0.82
Lewy body Dementia			Apply	Diagnosis	0.98	0.99
Average Score					0.87	0.71

^Values reported by the NBME. Asterisks (*) indicate the final diagnosis for the four SPEs; all other diagnoses represent differentials discussed. P-value cannot be calculated by question between groups, but average score on all selected questions was compared between groups with a Mann-Whitney U test, where *p* < 0.05

**Fig. 3:**
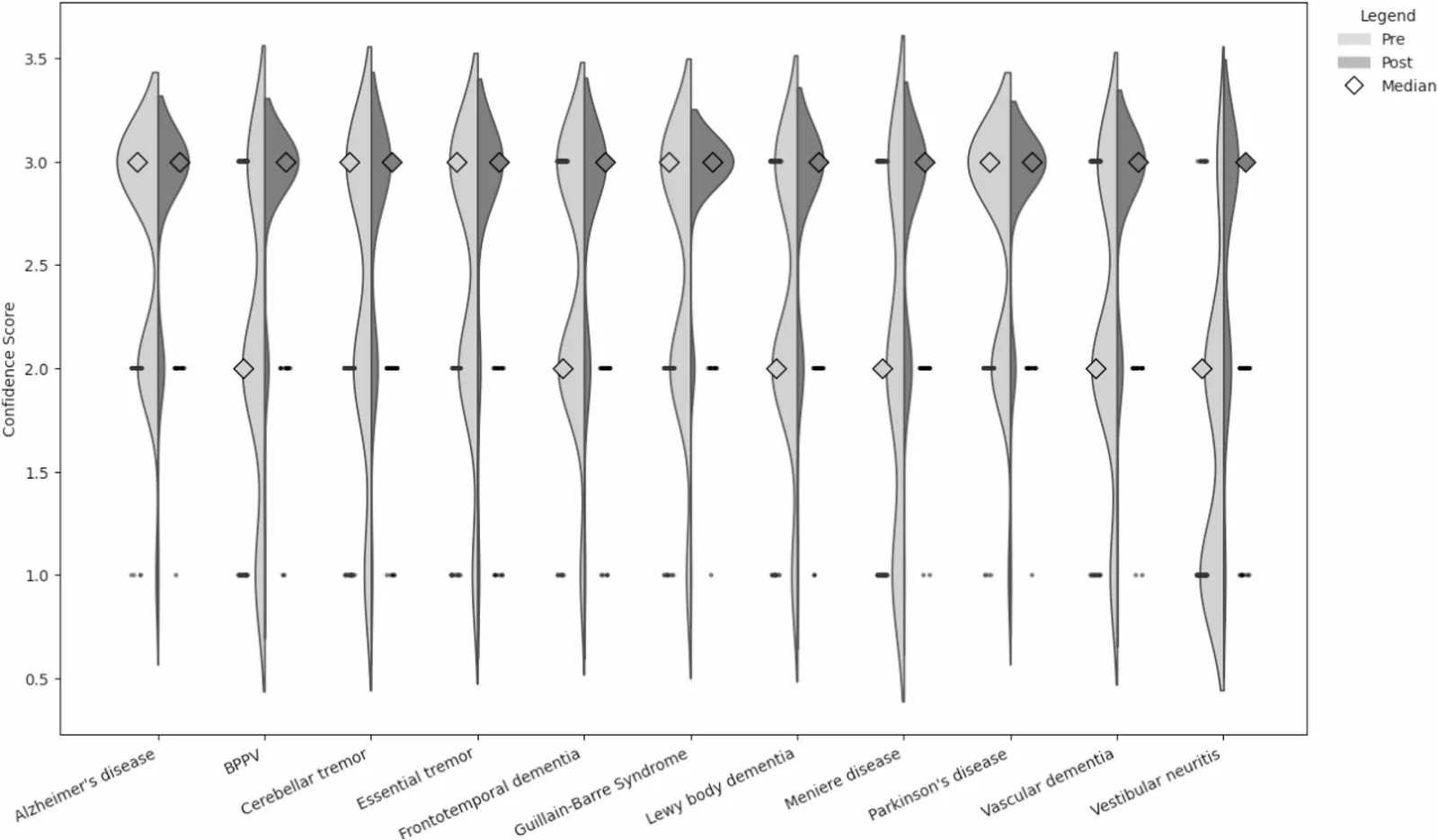
Distribution of pre- and post-standardized patient encounter (SPE) confidence scores across clinical diagnoses. Each violin represents the kernel-density distribution of scores for either the pre- or post-SPE assessment, with lighter gray indicating pre-SPE and darker gray indicating post-SPE. The width of each violin reflects the relative concentration of scores at a given value; therefore, although portions of the violin extend above the maximum possible score of 3 on the y-axis, this represents density contours rather than actual values. Asymmetry and long tails in the violins indicate skewed or dispersed scoring patterns within each item. Individual student scores are shown as black dots, illustrating paired observations. Black-outlined diamonds indicate the median score for each group. Overall, these distributions demonstrate statistically significant increases in confidence following the SPE across all items, confirmed with individual Wilcoxon signed-rank tests (all *P* < 0.001). Abbreviations. BPPV: Benign Paroxysmal Positional Vertigo

In parallel to the knowledge gain, students demonstrated statistically significant gains in confidence in their ability to diagnose all the covered conditions (Fig. 3). Participation in the SPEs was associated with significant increases in student confidence across all 11 neurological diagnoses assessed (all *p* < 0.001). Although formal qualitative analysis was not conducted, unsolicited student feedback from end-of-course evaluations reflected positive perceptions of the SPE experience, particularly highlighting its value in reinforcing clinical reasoning and improving engagement. Representative quotes (unsolicited) in the student survey included:

“I enjoyed the anatomy lab and the SP encounters.”

“Well organized, I like the flow of information. I particularly enjoyed the last session with the SP that showed different conditions, their presentation, and management.”

## Discussion

In this study, we demonstrated that incorporating SPEs into a pre-clerkship Nervous System course was associated with significant gains in confidence diagnosing targeted conditions, an immediate knowledge gain after the session, and enhanced performance in SPE-related questions in an NBME exam, compared to national average. To our knowledge, this is among the first reports to evaluate how SPEs in a pre-clerkship neurology curriculum can translate into superior performance on standardized exams. Prior studies have largely examined clerkship-level or residency cohorts, where SPE participation was associated with higher OSCE scores and improved clinical evaluations [5, 6, 11, 12, 19], but few studies extend these findings to earlier learning stages or NBME question outcomes. Our findings extend this evidence to the pre-clerkship setting. Integrating these experiences early in the curriculum is essential not only for reinforcing short-term clinical knowledge but also for building the foundational skills and confidence students need before their first patient encounters in the clerkships, when uncertainty and inexperience can undermine confidence and hinder learning.

Confidence gains observed in this study are meaningful beyond the performances on self-assessment. Previous literature has consistently shown that SPEs can enhance student self-confidence in areas such as communication, interviewing, and management of acute conditions [9, 17, 21]. Confidence is not only an important learner outcome in its own right, but has also been shown to correlate with improved performance in clinical settings [16]. Our findings are particularly meaningful given that the effect was observed in the pre-clerkship phase at an early stage of training. This increased confidence can encourage students to engage more fully with patients, rather than approaching their first encounters with hesitation or fear. Indeed, the higher confidence resulting from focused simulation correlates with higher competence at later stages [22].

Targeted sessions based on curricular deficiencies or needs can have a meaningful educational impact. In our case, the topics selected for the SPE intervention were based on prior student feedback after the course or during clerkship years, where certain areas were identified as challenging. For example, students had previously found vestibular disorders particularly challenging after the course, in part because of the lack of emphasis and clinical examples in class. Indeed, this was one of the areas of highest gain in confidence after the session. On the other hand, topics that were emphasized in prior curricular case-based learning sessions (i.e. Alzheimer’s and Parkinson’s Disease) had the least gain in confidence. Most of this difference was attributed to higher ratings before the intervention for these conditions (above 2.5), reflecting an initial sense of perceived understanding and mastering of the material, compared to lower rating for the other conditions. By proactively targeting topics that historically posed difficulty, this approach has the potential to enhance curriculum and ease the transition into the clerkship phase.

The increased confidence was matched with knowledge gains, as demonstrated by higher scores in a post-session quiz, compared to the pre-quiz. Most of the gains were observed in questions that had lower baseline values. This highly suggests that the session reinformed key concepts and addressed gaps present at the baseline. Notably, our students outperformed national averages in questions related to the diagnoses covered in the SPEs. To our knowledge, we are the first to show this increase in a pre-clerkship neurology course. Although the NBME comparison should be interpreted with caution and should not be viewed as the sole indicator of the SPEs’ effectiveness, national data is an important external benchmark that provides helpful background, highlighting areas of strength and opportunities for improvement. For example, despite the high confidence and high post-session quiz scores, one of the least improved NBME questions was related to Alzheimer’s disease. This could be due to the complexity of the question used or students overestimating their understanding of the material as they prepared for exams or answered questions. Therefore, it is important to incorporate targeted feedback to help students align their confidence with their true level of understanding.

There are some additional factors to consider before SPE implementation, including cost, resource intensity, and variability in long-term SP retention. We employed eight SPs for the 4-hour SPE session and required training, with compensation ranging from $20-$25 per hour based on experience, totaling $3,245. In a different study using SPEs to teach disease management through one case to 72 students, total costs were $4,400 [23]. Thus, cost-benefit analysis and scalability should be examined to guide broader curricular adoption.

### Limitations & Future Directions

While our study demonstrates promising outcomes, it is limited by its single-course and institution design. Nonetheless, we believe that our targeted approach, focusing on prior gaps of knowledge, can be incorporated into a variety of settings, making our findings generalizable to other pre-clerkship courses and medical education programs.

The knowledge gains reported here should be interpreted with caution based on our methodology design. Although we used identical pre- and post-session assessments to enable direct within-subject comparison and control for variation in question difficulty, this approach may have introduced a testing effect, whereby students preferentially attended to previously encountered items, potentially influencing both learning behaviors during the session and post-test performance. Additionally, we did not have an internal control for the final exam due to logistical constraints and ethical concerns. Instead, we compared the exam performance using national NBME data on SPE related questions. Although this benchmark offered standardization and external comparability across other institutions, there might be confounders that we were are unable to account for and therefore, we cannot establish a direct cause-effect relationship. 

Moreover, our assessments were conducted within the duration of a course, and we did not assess long-term retention or downstream outcomes. Future studies should evaluate longitudinal designs to evaluate long-term retention, including delayed post-testing and evaluation of clinical performance outcomes such as clerkship performance, shelf examination scores, or USMLE Step 2 performance. Yet, attribution of such outcomes to a single pre-clerkship intervention is inherently limited given the influence of subsequent clinical training. 

A study comparing SPE performance in residency based on exposure to SPE during medical school found that prior exposure to SPs did not appear to have a positive effect on subsequent performance unless similar or identical cases were used [24].

The integrated structure of the SPE sessions, which combined hands-on patient encounters with facilitated group discussions, precludes determination of the relative contribution of each component to the observed outcomes. Additionally, the use of a 3-point Likert scale to assess confidence limited the granularity of measurement and may not have captured more subtle shifts in learner perception. Future studies could incorporate more nuanced scales to better characterize changes in confidence and qualitative explorations (e.g., focus groups) to better understand how learners engage and isolate SPE individual elements to better understand their educational impact. Moreover, incorporating SPs’ reflections on areas in which students appear more confident, engaged or challenged could provide additional insights into the educational effectiveness of the intervention and refine curricular design.

## Conclusion

Embedded within an experiential framework, we designed an SPE session to address identified learner gaps and curricular expectations by focusing on historically challenging topics and providing opportunities to actively apply foundational knowledge in a simulated clinical encounter. Integrating SPEs into a pre-clerkship neurology curriculum produced measurable gains in confidence, immediate knowledge acquisition, and strong NBME exam performance. These findings underscore SPEs’ potential to strengthen clinical reasoning and better prepare students for patient-facing roles earlier in their training, bridging the gap between pre-clerkship and clerkship years. Future studies should evaluate long-term retention, subsequent performance outcomes in clerkships, and scalability across institutions. 

## Data Availability

Data and code are available upon request to the corresponding author.
